# Supplementary material for: From chiral laser pulses to femto- and attosecond electronic chirality flips in achiral molecules
Source: Nat Commun. 2024 Jan 17;15:565. doi: 10.1038/s41467-024-44807-0 (PMC10794217; doi:10.1038/s41467-024-44807-0)
Supplement: Supplementary file 1 — Supplementary Information [file 41467_2024_44807_MOESM1_ESM.pdf]

Supplementary Information for

From chiral laser pulses to femto- and attosecond electronic chirality flips in achiral molecules

Yunjiao Chen[1], Dietrich Haase[2], Jörn Manz[1,2,3,*], Huihui Wang[1,*], Yonggang Yang[1,3,*]

Affiliations:

[1]State Key Laboratory of Quantum Optics and Quantum Optics Devices, Institute of Laser Spectroscopy, Shanxi University, Taiyuan 030006, China

[2] Institut für Chemie und Biochemie, Freie Universität Berlin, 14195 Berlin, Germany

[3] Collaborative Innovation Center of Extreme Optics, Shanxi University, Taiyuan 030006, China.

*To whom correspondence should be addressed: jmanz@chemie.fu-berlin.de (J. M.), huihuiwang2019@sxu.edu.cn (H. W.), ygyang@sxu.edu.cn (Y. Y.)

Table of Contents

Supplementary Note 1 3

Supplementary Note 2 6

Supplementary Note 3 19

Supplementary Note 4 23

Supplementary Figure 1 3

Supplementary Figure 2 4

Supplementary Figure 3 5

Supplementary Figure 4 21

Supplementary Figure 5 25

Supplementary Figure 6 27

Supplementary Table 1 22

Supplementary References 28

Supplementary Note 1: Chirality flips in NaK in the $1\ ^1\Sigma^+ + 1\ ^1\Pi_{\pm1} + 2\ ^1\Pi_{\pm1}$ superposition states documented by snapshots of the electronic density

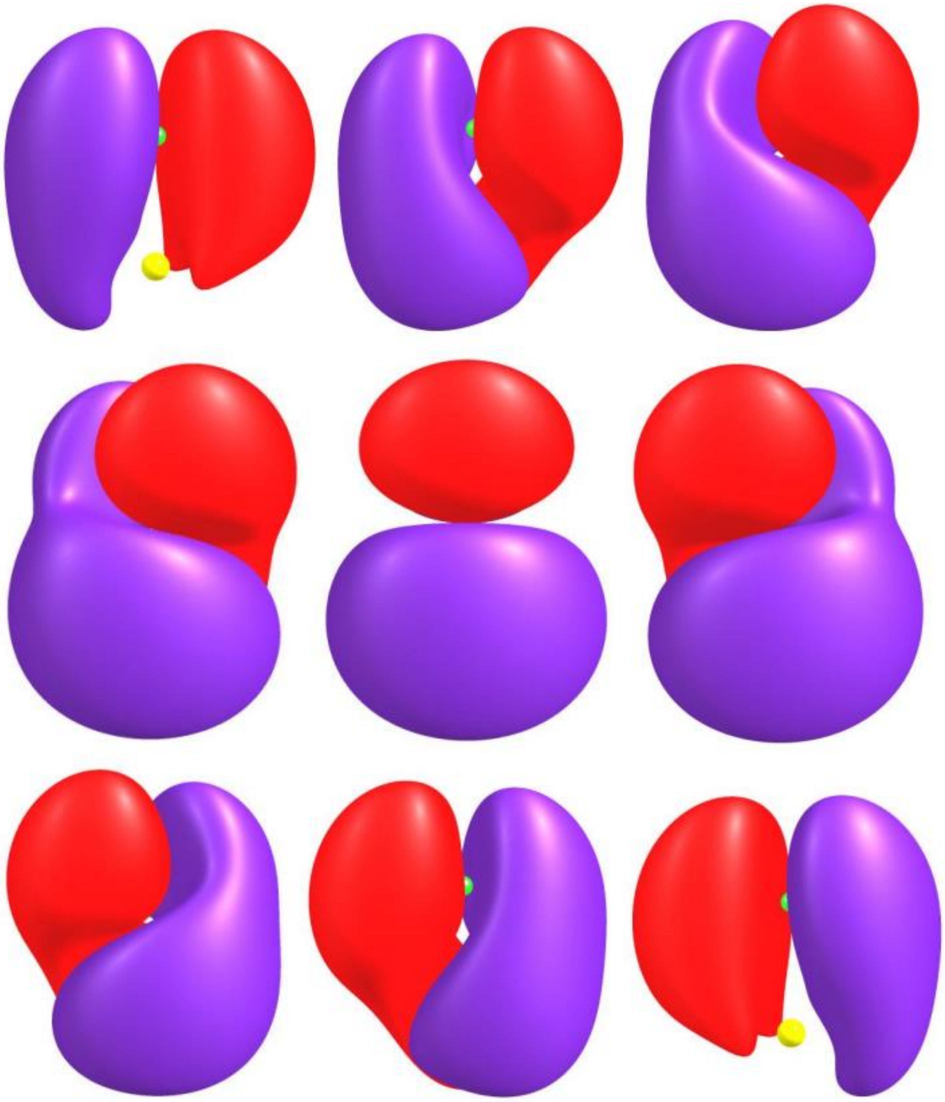

Supplementary Figure 1: Snapshots of the time-dependent part of the electronic density of NaK from $t = T_{0-+}$ to $t = T_{2-+}$ in steps of $\delta t = \frac{\Delta T_{-+}}{4}$, for the case of $(-+)$ circular polarizations of the laser pulses. All the figures are rotated around the *z*-axis by 49.2 degrees such that the middle panel, which is for $t = T_{1-+}$, has its symmetry plane perpendicular to the paper plane. The first row from left to right is for $t = T_{1-+} - 4\delta t = T_{0-+}$, $T_{1-+} - 3\delta t$, and $T_{1-+} - 2\delta t$. The middle row from left to right is for $t = T_{1-+} - \delta t$, T_{1-+}, and $T_{1-+} + \delta t$. The last row from left to right is for $t = T_{1-+} + 2\delta t$, $T_{1-+} + 3\delta t$, and $T_{1-+} + 4\delta t = T_{2-+}$.

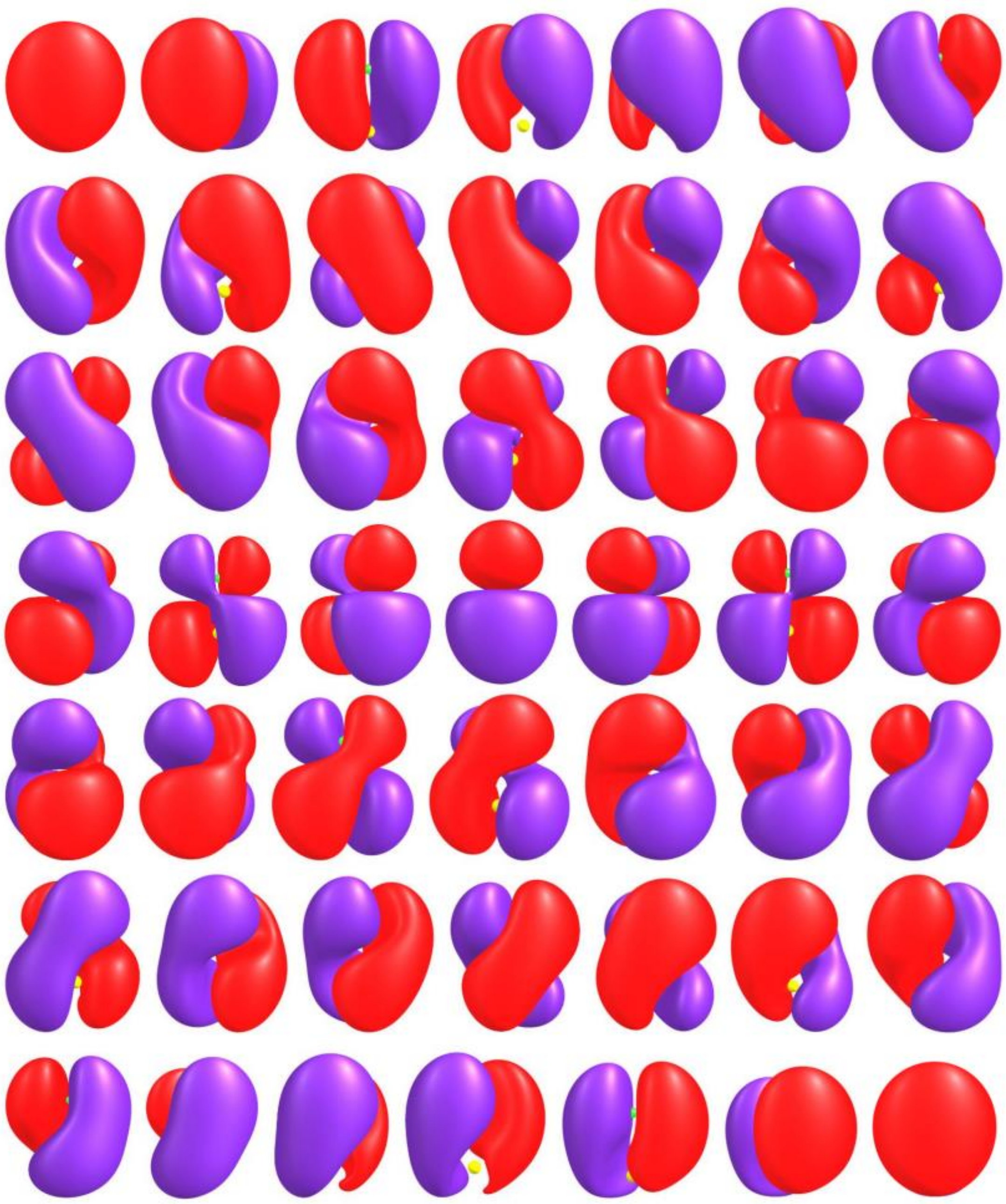

Supplementary Figure 2: Snapshots of the time-dependent part of the electronic density of NaK from $t = T_{0++}$ to $t = T_{2++}$ in steps of $\delta t = \frac{\Delta T_{++}}{24}$, for the case of $(++)$ circular polarizations of the laser pulses. All the figures are rotated around the *z*-axis by 188.0 degrees such that the middle panel, which is for $t = T_{1++}$, has its symmetry plane perpendicular to the paper plane. Similar to Supplementary Figure 1, the times of neighboring snapshots in each row from left to right increase by δt, while in each column from top to bottom they increase by $7\,\delta t$.

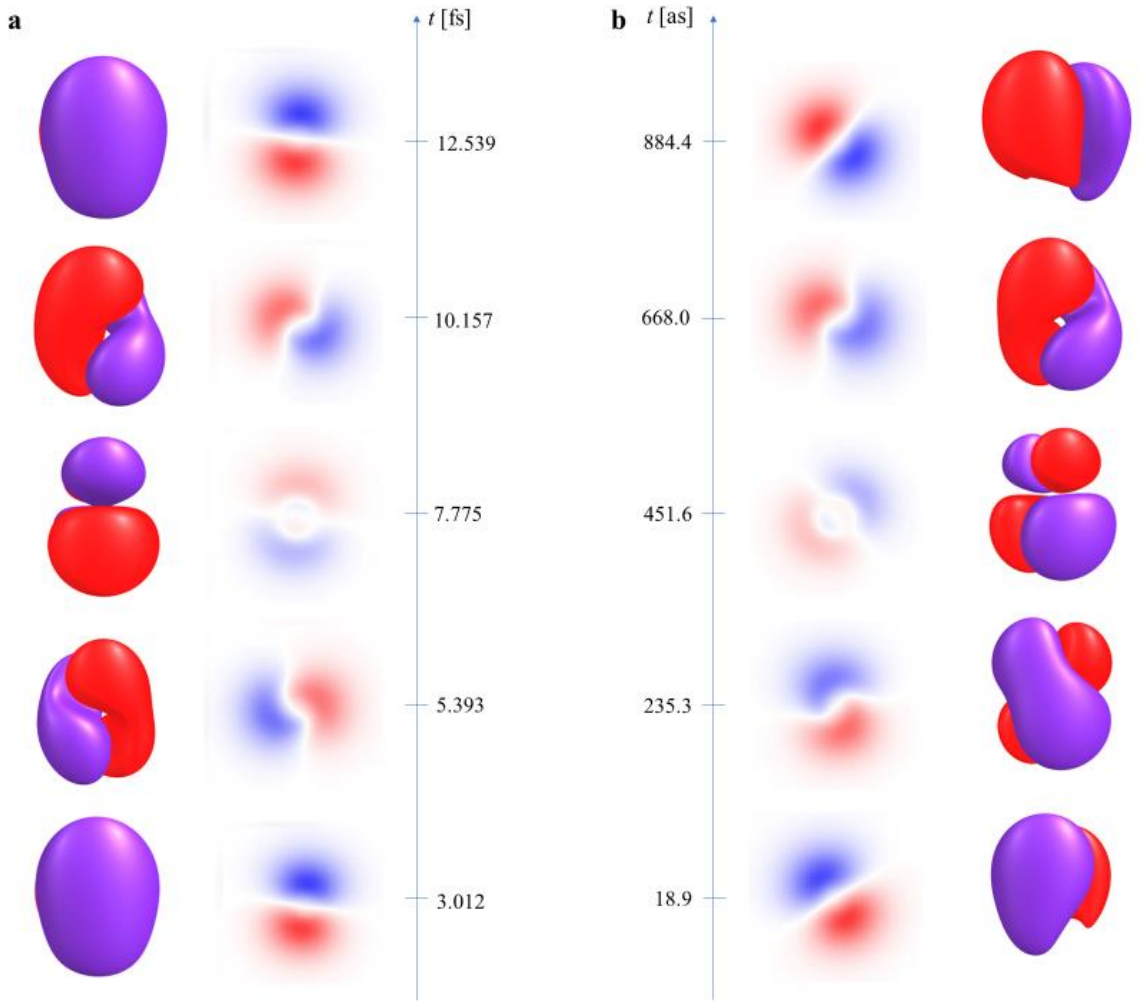

Supplementary Figure 3: Non-rotated version for the snapshots shown in Fig. 2 of the main text with corresponding two-dimensional reduced density $\rho(x,y,t)$**.** The times of the five snapshots are the same as for Fig. 2 of the main text.

The main text already documents chirality flips in NaK in the $1\ ^1\Sigma^+ + 1\ ^1\Pi_{\pm1} + 2\ ^1\Pi_{\pm1}$ superposition states by selective snapshots of the electronic density, cf. Fig. 2 of the main text. Here we support the documentation by additional snapshots in Supplementary Figures 1-3. Note for better view of the chirality flips, the snapshots in Fig. 2 of the main text are rotated around the z-axis by certain angles. Specifically, the five snapshots from top to bottom in Fig. 2a of the main text are rotated by 188.7, 188.0-30, 188.0, 188.0+30, and 187.3 degrees, respectively, while the five snapshots from top to bottom in Fig. 2b of the main text are rotated by 147.4, 49.2, 49.2, 49.2,

and -49.0 degrees, respectively. To show all snapshots in the same coordinates frame, we plot snapshots that are all rotated around the *z*-axis by the same angle in Supplementary Figures 1 and 2. Finally, we show snapshots in the original coordinates frame, namely none of the snapshots is rotated, in Supplementary Figure 3, together with the corresponding snapshots of the two-dimensional reduced density

$$\rho_{\pm\pm}(x,y,t) = \int_{-\infty}^{+\infty} \rho_{\pm\pm}(r,z,\phi,t)dz \,. \tag{S1}$$

Supplementary Note 2: Derivation of equation (2) of the main text for the time evolution of the electronic density of an oriented heteronuclear diatomic molecule such as NaK prepared in the $1\ ^1\Sigma^+ + 1\ ^1\Pi_{\pm1} + 2\ ^1\Pi_{\pm1}$ superposition states.

Our step-by-step presentation in this section reminds of various properties of the underlying electronic Hamilton operator, and of the electronic eigenfunctions, the many-electron probability density, and the electronic density at the level of full configuration interaction (full-CI). The final result eqn. (S66), will be obtained at the end of this Section.

2.1: Molecular orbitals of oriented heteronuclear diatomic molecules

We consider heteronuclear diatomic molecules AB such as NaK with N electrons. The molecules are oriented along the laboratory *z*-axis, with nucleus A pointing to positive values of *z*. The nuclear center of mass is at the origin. The nuclei have cylindrical symmetry $C_{\infty\mathrm{v}}$. This suggests to use cylindrical coordinates $\mathbf{r} = (r,z,\phi)$ for the electronic positions. The corresponding combined set of spatial and electron spin coordinates is written as $\mathbf{q} = (\mathbf{r},s)$.

The corresponding molecular orbitals (MOs) $\psi_{mn}(r,z,\phi)$ are eigenfunctions of the one-electron Hamiltonian

$$H^{(1)}(\mathbf{r}) = T^{(1)}(\mathbf{r}) + V^{(1)}(\mathbf{r};R) \tag{S2}$$

where $T^{(1)}(\mathbf{r})$ and $V^{(1)}(\mathbf{r};R)$ are the operators of the kinetic and potential energies of the electron,

$$T^{(1)}(\mathbf{r}) = -(\frac{\hbar^2}{2m_\mathrm{e}})(\frac{1}{r}\frac{\partial}{\partial r}r\frac{\partial}{\partial r}) - (\frac{\hbar^2}{2m_\mathrm{e}})(\frac{\partial^2}{\partial z^2}) - (\frac{\hbar^2}{2m_\mathrm{e}r^2})(\frac{\partial^2}{\partial \phi^2}) \tag{S3}$$

with electron mass m_e. The potential energy $V^{(1)}(\mathbf{r};R)$ is approximated as the sum of the Coulomb interactions of the electron with the two nuclei. Interactions of nuclear and electronic spins are neglected.

The Hamiltonian (S2) commutes with the *z*-component of the electron's angular momentum operator,

$$\left[H^{(1)}, l_z\right] = 0 , \tag{S4}$$

$$l_z = -i\hbar\partial / \partial\phi . \tag{S5}$$

As a consequence, the MOs are not only eigenfunctions of $H^{(1)}$, but also of l_z,

$$l_z\psi_{mn}(r,z,\phi) = m\hbar\psi_{mn}(r,z,\phi) , \qquad m = 0,\ \pm 1,\ \pm 2,\ ... \tag{S6}$$

$$H^{(1)}\psi_{mn}(r,z,\phi) = e_{mn}\psi_{mn}(r,z,\phi) , \qquad n = 1,\ 2,\ ... \tag{S7}$$

with MO angular momentum $m\hbar$ and MO energy e_{mn}.

Hence the MOs take the form

$$\psi_{mn}(r,z,\phi) = \chi_{mn}(r,z)\frac{1}{\sqrt{2\pi}}e^{im\phi} \tag{S8}$$

with

$$\chi_{mn} = \chi_{-mn} = \chi_{|m|n} .$$

The corresponding orbital densities

$$\rho_{mn}(r,z) = \psi^*_{mn}(r,z,\phi)\psi_{mn}(r,z,\phi) = \chi^2_{mn}(r,z) \tag{S9}$$

are independent of the angle ϕ, i. e. they adapt the cylindrical symmetry $C_{\infty v}$ of the nuclei. The densities of the degenerate MOs with angular momentum quantum numbers m and $-m$ are equal to each other,

$$\rho_{-mn}(r,z) = \rho_{mn}(r,z) . \tag{S10}$$

The cylindrically symmetric (i. e. independent of ϕ) real-valued functions $\chi_{mn}(r,z)$ are solutions of the Schrödinger equation

$$\left[-(\frac{\hbar^2}{2m_e})(\frac{1}{r}\frac{\partial}{\partial r}r\frac{\partial}{\partial r}) - (\frac{\hbar^2}{2m_e})(\frac{\partial^2}{\partial z^2}) - (\frac{m^2\hbar^2}{2m_e r^2})\right]\chi_{mn}(r,z) = e_{mn}\chi_{mn}(r,z) . \tag{S11}$$

Exact analytical forms of $\chi_{mn}(r,z)$ are reported in (Ref. 1). The corresponding molecular spin-orbitals for electrons with α or β spins are written as

$$\varphi_{mnm_s}(\mathbf{q}) = \chi_{mn}(r,z)\frac{1}{\sqrt{2\pi}}e^{-im\phi}\sigma_{m_s}(s) \equiv \varphi_l(\mathbf{q}) \tag{S12}$$

where $\sigma_{m_s=1/2}(s) = \alpha(s)$ and $\sigma_{m_s=-1/2}(s) = \beta(s)$, respectively. Spin-orbitals with opposite quantum numbers m and $-m$ are degenerate and complex conjugate to each other,

$$\varphi_{-mnm_s}(\mathbf{q}) = \varphi^*_{mnm_s}(\mathbf{q}) \ . \tag{S13}$$

The set of quantum numbers (m,n,m_s) is mapped on the label l in energetic order for (m,n) and with alternating spin and angular momentum quantum numbers, e. g.

$$\begin{aligned}(m,\ n,\ m_s) &= (0,\ 1,\ 1/2) &&\rightarrow\ l=1\ ,\\ &= (0,\ 1,\ -1/2) &&\rightarrow\ l=2\ ,\\ &= (1,\ 1,\ 1/2) &&\rightarrow\ l=3\ ,\\ &= (1,\ 1,\ -1/2) &&\rightarrow\ l=4\ ,\\ &= (-1,\ 1,\ 1/2) &&\rightarrow\ l=5\ ,\ \text{etc.}\end{aligned}$$

The molecular spin-orbitals are normalized and orthogonal. In shorthand Dirac notation,

$$\begin{aligned}\langle \varphi_l \mid \varphi_{l'} \rangle &= \int \varphi_l^*(\mathbf{q})\varphi_{l'}(\mathbf{q})d\mathbf{q}\\ &= \int_0^\infty rdr\int_{-\infty}^\infty dz\chi_{mn}(r,z)\chi_{m'n'}(r,z)\frac{1}{2\pi}\int_0^{2\pi} d\phi e^{i(m'-m)\phi}\int ds\sigma_{m_s}(s)\sigma_{m'_s}(s)\\ &= \delta_{ll'} = \delta_{mm'}\delta_{nn'}\delta_{m_s,m'_s} \ .\end{aligned} \tag{S14}$$

2.2: Slater determinants for oriented heteronuclear diatomic molecules

The electronic eigenfunctions of oriented heteronuclear molecules AB such as NaK with N electrons are described in terms of Slater determinants for N molecular spin-orbitals $\varphi_{l_1}, \varphi_{l_2}, ..., \varphi_{l_N}$. By convention, they are listed with ascending order of the labels for the quantum numbers, $l_1 < l_2 < ... < l_N$. In shorthand notation

$$| \varphi_{l_1}, \varphi_{l_2}, ..., \varphi_{l_N} | = \sqrt{N!}\mathcal{A}\varphi_{l_1}\varphi_{l_2}...\varphi_{l_N} \tag{S15}$$

where $\mathcal{A}$ is the anti-symmetrization operator,

$$\mathcal{A} = (1/N!)\sum_{\nu=1}^{N!}(-1)^{p_\nu}P_\nu = (1/N!)(1+\sum_{\nu=2}^{N!}{}'(-1)^{p_\nu}P_\nu) \tag{S16}$$

with normalized sum of all even ($(-1)^{P_v} = +1$) and odd ($(-1)^{P_v} = -1$) permutations of the molecular spin-orbitals; the sum Σ' is over all permutations except the identity ("1"). The relation (S13) for the molecular spin-orbitals implies the complex conjugation of the Slater determinants,

$$|\varphi_{l_1}, \varphi_{l_2}, ..., \varphi_{l_N}|^* = |\varphi_{l_1}^*, \varphi_{l_2}^*, ..., \varphi_{l_N}^*| \quad . \tag{S17}$$

Slater-determinants are normalized, and Slater-determinants for different sets of molecular spin-orbitals are orthogonal. The proof is in the textbooks. Nevertheless, we remind of it because analogous steps will be used for the subsequent derivation of the one-electron densities, which is non-standard. In short-hand Dirac notation,

$$\begin{aligned}&\left\langle l_1, l_2, ..., l_N \middle| l_1^{'}, l_2^{'}, ..., l_N^{'} \right\rangle \\ &= N! \int ... \int d\mathbf{q}_1 ... d\mathbf{q}_N \left[\mathcal{A}\varphi_{l_1}^*(\mathbf{q}_1) ... \varphi_{l_N}^*(\mathbf{q}_N) \right] \times \left[\mathcal{A}\varphi_{l_1'}(\mathbf{q}_1) ... \varphi_{l_N'}(\mathbf{q}_N) \right] \\ &= N! \int ... \int d\mathbf{q}_1 ... d\mathbf{q}_N \varphi_{l_1}^*(\mathbf{q}_1) ... \varphi_{l_N}^*(\mathbf{q}_N) \mathcal{A}\varphi_{l_1'}(\mathbf{q}_1) ... \varphi_{l_N'}(\mathbf{q}_N) \ .\end{aligned} \tag{S18}$$

The second eqn. (S18) exploits the fact that $\mathcal{A}$ is Hermitian and a projection operator,

$$\mathcal{A}^2 = \mathcal{A} = \mathcal{A}^\dagger \ . \tag{S19}$$

The overlap integral (S18) is equal to zero if the two sets of spin-orbitals labeled $l_1, ..., l_N$ and $l_1^{'}, ..., l_N^{'}$ differ from each other, due to the orthogonality of the molecular spin-orbitals (S14). For equal sets of spin-orbitals, eqn. (S18) is evaluated by means of eqn. (S16),

$$\begin{aligned}\left\langle l_1, l_2, ..., l_N \middle| l_1, l_2, ..., l_N \right\rangle &= N! \int ... \int d\mathbf{q}_1 ... d\mathbf{q}_N \varphi_{l_1}^*(\mathbf{q}_1) ... \varphi_{l_N}^*(\mathbf{q}_N)(1/N!) \\ &\left[\varphi_{l_1}(\mathbf{q}_1) ... \varphi_{l_N}(\mathbf{q}_N) + \sum_{v=2}^{N!}{}' (-1)^{P_v} P_v \varphi_{l_1}(\mathbf{q}_1) ... \varphi_{l_N}(\mathbf{q}_N) \right] \\ &= 1 + \sum_{v=2}^{N!}{}' 0 = 1 \ .\end{aligned} \tag{S20}$$

The sum $\sum_{v=2}^{N!}{}'$ does not contribute, due to the orthogonality of the spin-orbitals. To summarize, Slater determinants are orthonormal,

$$\left\langle l_1, l_2, ..., l_N \middle| l_1^{'}, l_2^{'}, ..., l_N^{'} \right\rangle = \delta_{l_1, l_1^{'}} \delta_{l_2, l_2^{'}} ... \delta_{l_N, l_N^{'}} \quad . \tag{S21}$$

2.3: Electronic eigenfunctions for the $1\ \Sigma^{+}$, $1\ \Pi_{\pm 1}$ and $2\ \Pi_{\pm 1}$ states of oriented heteronuclear diatomic molecules

Electronic eigenfunctions of oriented heteronuclear diatomic molecules AB such as NaK are obtained as solutions of the electronic Schrödinger equation

$$H_{\mathrm{el}} \Psi_k = E_k \Psi_k \, . \tag{S22}$$

In analogy to the molecular spin-orbitals $\varphi_l(\mathbf{q})$, we use the label k for the adequate set of quantum numbers.

The electronic Hamilton operator can be written as

$$H_{\mathrm{el}} = \sum_{j=1}^{N} H^{(1)}(\mathbf{r}_j) + \sum_{i<j} V_{\mathrm{el,el}}\left(\left|\mathbf{r}_i - \mathbf{r}_j\right|\right) + V_{\mathrm{nu,nu}}(R) \tag{S23}$$

with Coulomb interactions between the electrons labeled i and j,

$$\begin{aligned} V_{\mathrm{el,el}}\left(\left|\mathbf{r}_i - \mathbf{r}_j\right|\right) &= e^2 / \left(4\pi\varepsilon_0 \left|\mathbf{r}_i - \mathbf{r}_j\right|\right) \\ &= e^2 / \left(4\pi\varepsilon_0 \sqrt{\left[r_i^2 + 2 r_i r_j \cos(\phi_i - \phi_j) + r_j^{\,2} + (z_i - z_j)^2 \right]}\right) \end{aligned} \tag{S24}$$

and the Coulomb interaction $V_{\mathrm{nu,nu}}(R) = \dfrac{Q_A Q_B e^2}{4\pi\varepsilon_0 R}$ between the two nuclei with charges $Q_A e$ and $Q_B e$ at distance R. It commutes with the z-component of the angular momentum operator

$$L_z = \sum_{j=1}^{N} l_{z,j} = \sum_{j=1}^{N} \left(-i\hbar \partial / \partial \phi_j\right) , \tag{S25}$$

$$\left[H_{\mathrm{el}}, L_z \right] = 0 \, . \tag{S26}$$

The electronic eigenfunctions are, therefore, not only eigenfunctions of the electronic Hamilton operator, eqn. (S22), but also of L_z,

$$L_z \Psi_k = M\hbar \Psi_k, \ M = 0,\ \pm 1,\ \pm 2, ... \tag{S27}$$

As a consequence, the wavefunctions Ψ_k can be written in full configuration interaction (full-CI) as sums of Slater determinants

$$\Psi_k = \sum_{l_1 < ... < l_N} C^{k}_{l_1,...,l_N} \left| \varphi_{l_1}, \varphi_{l_2}, ..., \varphi_{l_N} \right| \tag{S28}$$

subject to the condition that the angular momentum quantum numbers m_j of the spin-orbitals φ_{l_j} must sum up to the chosen value of M,

$$\sum_j m_j = \mathrm{M}. \tag{S29}$$

The coefficients $C^k_{l_1,\dots,l_N}$ are real-valued. Since the electronic Hamiltonian does not depend on electron spins, it also commutes with the electron spin operators. The total electron spin S and its *z*-component S_z yield, therefore, also good quantum numbers. In particular, the magnetic spin quantum numbers $m_{s,j}$ must sum up to the chosen total value $\mathrm{M_s}$,

$$\sum_j m_{s,j} = \mathrm{M_s}. \tag{S30}$$

In the present application, we focus on singlet states ($S=0$, $\mathrm{M_s}=0$). Eqn. (S30) then implies that for even numbers N of electrons, the numbers of α and β spin-orbitals in the Slater determinants are equal to each other.

The present application focusses on the ground state $1\ ^1\Sigma^+$ and two pairs of degenerate excited states $1\ ^1\Pi_{+1}$, $1\ ^1\Pi_{-1}$ as well as $2\ ^1\Pi_{+1}$, $2\ ^1\Pi_{-1}$. These have quantum numbers $k=(M,N_E,M_s)=(0,1,0)$ and (1,1,0), (-1,1,0) as well as (1,2,0), (-1,2,0), respectively. Here N_E is the quantum number for different values of energies. For convenience, the five states are labeled $k=0$ and $k=1+,\ 1-$ as well as $k=2+,\ 2-$, respectively. The energy of the ground state is set equal to zero, $E_{k=0}\equiv 0$. The energies of the excited states are degenerate; they are written E_1 as well as E_2. Accordingly, the full-CI expansion for the $1\ ^1\Sigma^+$ state ($k=0$) is denoted

$$\Psi_{k=0} = \sum_{l_1<\dots<l_N} C^0_{l_1,\dots,l_N} \left|\varphi_{l_1},\varphi_{l_2},\dots,\varphi_{l_N}\right| \text{ with } \sum_j m_j = 0, \tag{S31}$$

and the expansions for the degenerate states $1\ ^1\Pi^\pm$ ($k=\pm1$) and $2\ ^1\Pi^\pm$ ($k=\pm2$) are

$$\Psi_{1\pm} = \sum_{l_1<\dots<l_N} C^{1\pm}_{l_1,\dots,l_N} \left|\varphi_{l_1},\varphi_{l_2},\dots,\varphi_{l_N}\right| \text{ with } \sum_j m_j = \pm1, \tag{S32}$$

$$\Psi_{2\pm} = \sum_{l_1<\dots<l_N} C^{2\pm}_{l_1,\dots,l_N} \left|\varphi_{l_1},\varphi_{l_2},\dots,\varphi_{l_N}\right| \text{ with } \sum_j m_j = \pm1. \tag{S33}$$

The degenerate wavefunctions are complex conjugate to each other, in accord with the complex conjugation of the spin-orbitals and the Slater determinants with opposite angular momentum quantum numbers, cf. eqns. (S13), (S17)

$$\Psi_{1-} = \Psi^*_{1+}, \qquad \Psi_{2-} = \Psi^*_{2+}. \tag{S34}$$

The orthonormality of the Slater determinants implies that the electronic eigenfunctions (S31)-(S33) are normalized according to

$$\left\langle \Psi_k \middle| \Psi_k \right\rangle = \sum_{l_1<...<l_N} \left| C^k_{l_1,...,l_N} \right|^2 = 1, \tag{S35}$$

and they are also orthogonal,

$$\left\langle \Psi_k \middle| \Psi_{k'} \right\rangle = \delta_{k,k'} \,. \tag{S36}$$

2.4: Many-electron probability density and electronic density of the electronic eigenfunctions of the Σ^+, $1\ \Pi_{\pm 1}$ and $2\ \Pi_{\pm 1}$ states of oriented heteronuclear diatomic molecules

Many-electron probability densities of the full-CI eigenfunctions (S31)-(S33) are

$$\rho_k^{(N)} = \Psi_k^* \Psi_k = \sum_{l_1<...<l_N} C^k_{l_1,...,l_N} \left| \varphi_{l_1}, \varphi_{l_2}, ..., \varphi_{l_N} \right|^* \sum_{l_1'<...<l_N'} C^k_{l_1',...,l_N'} \left| \varphi_{l_1'}, \varphi_{l_2'}, ..., \varphi_{l_N'} \right| . \tag{S37}$$

The complex conjugation of the degenerate eigenfunctions (S34) implies that they have the same many-electron probability densities,

$$\rho_{1-}^{(N)} = \rho_{1+}^{(N)} , \qquad \rho_{2-}^{(N)} = \rho_{2+}^{(N)} \,. \tag{S38}$$

The expressions for the many-electron densities, eqn. (S37) at the full-CI level are formidable. We shall now show, however, that they yield rather simple results for the related electronic densities. For a quantum state $\left| \Psi_k \right\rangle$, the electronic density at the position $\mathbf{r}$ can be obtained as the mean value of the density operator $\hat{\rho}(\mathbf{r})$

$$\hat{\rho}(\mathbf{r}) = \sum_{j=1}^{N} \delta(\mathbf{r}_j - \mathbf{r}) \,. \tag{S39}$$

The resulting electronic densities for $k = 0$, $1\pm$ and $2\pm$ are

$$\begin{aligned} \rho_k(\mathbf{r}) &= \left\langle \Psi_k \middle| \hat{\rho}(\mathbf{r}) \middle| \Psi_k \right\rangle \\ &= \sum_{j=1}^{N} \sum_{l_1<...<l_N} \sum_{l_1'<...<l_N'} C^k_{l_1',...,l_N'} C^k_{l_1,...,l_N} \\ &\int ... \int d\mathbf{q}_1 ... d\mathbf{q}_N \left| \varphi_{l_1}, \varphi_{l_2}, ..., \varphi_{l_N} \right|^* \delta(\mathbf{r}_j - \mathbf{r}) \left| \varphi_{l_1'}, \varphi_{l_2'}, ..., \varphi_{l_N'} \right| \,. \end{aligned} \tag{S40}$$

Let us now consider the integrals in expression (S40). Using the anti-symmetrization operator $\mathcal{A}$ as in eqns. (S18)-(S20), we obtain

$$\begin{aligned}
&\int ...\int d\mathbf{q}_1...d\mathbf{q}_N \left|\varphi_{l_1},\varphi_{l_2},...,\varphi_{l_N}\right|^* \delta(\mathbf{r}_j-\mathbf{r})\left|\varphi_{l_1^{'}},\varphi_{l_2^{'}},...,\varphi_{l_N^{'}}\right| \\
&= N!\int ...\int d\mathbf{q}_1...d\mathbf{q}_N \varphi^*_{l_1}(\mathbf{q}_1)...\varphi^*_{l_N}(\mathbf{q}_N)\delta(\mathbf{r}_j-\mathbf{r})\mathcal{A}\varphi_{l_1^{'}}(\mathbf{q}_1)..\varphi_{l_N^{'}}(\mathbf{q}_N) \\
&= \int ...\int d\mathbf{q}_1...d\mathbf{q}_N \varphi^*_{l_1}(\mathbf{q}_1)...\varphi^*_{l_N}(\mathbf{q}_N)\delta(\mathbf{r}_j-\mathbf{r})[\varphi_{l_1^{'}}(\mathbf{q}_1)..\varphi_{l_N^{'}}(\mathbf{q}_N) \\
&+\sum_{\nu=2}^{N!}{}'(-1)^{P_\nu}P_\nu\varphi_{l_1^{'}}(\mathbf{q}_1)...\varphi_{l_N^{'}}(\mathbf{q}_N)]\ .
\end{aligned} \tag{S41}$$

The orthonormality of the spin-orbitals implies that the set of orbitals $\varphi_{l_1},...,\varphi_{l_N}$ must be the same as $\varphi_{l_1^{'}},...,\varphi_{l_N^{'}}$, except for the orbitals φ_{l_j} and $\varphi_{l_j^{'}}$,

$$\varphi_{l_1^{'}},...,\varphi_{l_{j-1}^{'}},...,\varphi_{l_{j+1}^{'}},...,\varphi_{l_N^{'}} = \varphi_{l_1},...,\varphi_{l_{j-1}},\varphi_{l_{j+1}},...,\varphi_{l_N}\ . \tag{S42}$$

Moreover, any permutations P_v of the orbitals $\varphi_{l_1^{'}}(\mathbf{q}_1)...\varphi_{l_N^{'}}(\mathbf{q}_N)$ would yield zero integrals in expression (S41), again due to the orthonormality of the spin orbitals. Hence, we are left with

$$\begin{aligned}
&\int ...\int d\mathbf{q}_1...d\mathbf{q}_N \left|\varphi_{l_1},\varphi_{l_2},...,\varphi_{l_N}\right|^* \delta(\mathbf{r}_j-\mathbf{r})\left|\varphi_{l_1^{'}},\varphi_{l_2^{'}},...,\varphi_{l_N^{'}}\right| \\
&= \int ...\int d\mathbf{q}_1...d\mathbf{q}_N \varphi^*_{l_1}(\mathbf{q}_1)...\varphi^*_{l_N}(\mathbf{q}_N)\delta(\mathbf{r}_j-\mathbf{r})\varphi_{l_1^{'}}(\mathbf{q}_1)..\varphi_{l_N^{'}}(\mathbf{q}_N) \\
&= \int \varphi^*_{l_j}(\mathbf{q}_j)\delta(\mathbf{r}_j-\mathbf{r})\varphi_{l_1^{'}}(\mathbf{q}_j)d\mathbf{q}_j\ .
\end{aligned} \tag{S43}$$

The equality of all spin-orbitals but one, eqn. (S42) implies that the corresponding angular orbital quantum numbers must also be the same,

$$m_1^{'},m_2^{'},...,m_{j-1}^{'},m_{j+1}^{'},...,m_N^{'} = m_1,m_2,...,m_{j-1},m_{j+1},...,m_N\ . \tag{S44}$$

Likewise, the corresponding spin quantum numbers must be equal to each other,

$$m_{s,1}^{'},\ m_{s,2}^{'},\ ...,\ m_{s,j-1}^{'},\ m_{s,j+1}^{'},\ ...,\ m_{s,N}^{'} = m_{s,1},\ m_{s,2},\ ...,\ m_{s,j-1},\ m_{s,j+1},\ ...,\ m_{s,N}\ . \tag{S45}$$

Combining eqns. (S44), (S45) with the equalities (S29) and (S30) of the sums of all angular orbital and spin quantum numbers

$$\sum_j m_j^{'} = \sum_j m_j\ , \tag{S46}$$

$$\sum_j m_{s,j}^{'} = \sum_j m_{s,j}\ , \tag{S47}$$

implies that the angular momentum and spin quantum numbers of the spin-orbitals $\varphi_{l_j}(\mathbf{q}_j)$ and $\varphi_{l_j^{'}}(\mathbf{q}_j)$ must also be the same,

$$m_j^{'} = m_j,\ m_{s,j}^{'} = m_{s,j}\ . \tag{S48}$$

Inserting the explicit forms of the spin-orbitals (S12) then yields

$$\begin{aligned}
&\int ...\int d\mathbf{q}_1...d\mathbf{q}_N \left|\varphi_{l_1},\varphi_{l_2},...,\varphi_{l_N}\right|^* \delta(\mathbf{r}_j-\mathbf{r})\left|\varphi_{l_1'},\varphi_{l_2'},...,\varphi_{l_N'}\right| \\
&=\int d\mathbf{q}_j \psi^*_{m_j n_j}(r_j,z_j,\phi_j)\sigma_{m_s,j}(s_j)\delta(\mathbf{r}_j-\mathbf{r})\psi_{m_j n_j'}(r_j,z_j,\phi_j)\sigma_{m_s,j}(s_j) \\
&=\psi^*_{m_j n_j}(r,z,\phi)\psi_{m_j n_j'}(r,z,\phi) \\
&=\frac{1}{2\pi}\chi_{m_j n_j}(r,z)\chi_{m_j n_j'}(r,z).
\end{aligned} \tag{S49}$$

The electronic densities are thus evaluated as

$$\rho_k(\mathbf{r})=\frac{1}{2\pi}\sum_{j=1}^{N}\sum_{l_1<...<l_N}\sum_{l_1'<...<l_N'} C^k_{l_1',...,l_N'} C^k_{l_1,...,l_N}\delta_{l_1,l_1'}...\delta_{l_{j-1},l_{j-1}'}\delta_{l_{j+1},l_{j+1}'}...\delta_{l_N,l_N'}\chi_{m_j,n_j}(r,z)\chi_{m_j,n_j'}(r,z)\ . \tag{S50}$$

The result (S50) confirms chemical intuition that means the electronic densities of the eigenstates $1\ \Sigma^+$ and $1\ ^1\Pi_{\pm1}$, $2\ ^1\Pi_{\pm1}$ have cylindrical symmetry $C_{\infty v}$ – they do not depend on ϕ. Moreover, the equivalence (S38) implies that the electronic densities of the degenerate states $k=1\pm$ and $k=2\pm$ are equal to each other,

$$\rho_{1-}(\mathbf{r})=\rho_{1+}(\mathbf{r}), \tag{S51}$$

$$\rho_{2-}(\mathbf{r})=\rho_{2+}(\mathbf{r})\ . \tag{S52}$$

The total number of electrons can be obtained by the integration of the electronic density,

$$N=\frac{1}{2\pi}\int_0^\infty rdr\int_{-\infty}^{\infty}dz\int_0^{2\pi}d\phi\rho_k(r,z,\phi)\ . \tag{S53}$$

For the present application, the electronic densities $\rho_k(r)$, $k=0$, $1\pm$, $2\pm$ of NaK are illustrated in Fig. 1 of the main text. Analogous results can be derived for all eigenstates of oriented heteronuclear diatomic molecules.

2.5: The time-dependent electronic wave functions and the many-electron probability densities and the electronic densities of the $1\ ^1\Sigma^+ + 1\ ^1\Pi_{\pm1} + 2\ ^1\Pi_{\pm1}$ superposition states

In the present application, two circularly right (+) or left (–) polarized laser pulses with frequencies $\omega_1=E_1/\hbar$ and $\omega_2=E_2/\hbar$ prepare the $1\ ^1\Sigma^+ + 1\ ^1\Pi_{\pm1} + 2\ ^1\Pi_{\pm1}$ superposition states

$$\begin{aligned}
\Psi_{\pm\pm}(t)&=c_0\Psi_0+c_{1\pm}\Psi_{1\pm}e^{-i\omega_1 t}+c_{2\pm}\Psi_{2\pm}e^{-i\omega_2 t} \\
&=d_0\Psi_0+d_{1\pm}\Psi_{1\pm}e^{-i(\delta_1+\omega_1 t)}+d_{2\pm}\Psi_{2\pm}e^{-i(\delta_2+\omega_2 t)}
\end{aligned} \tag{S54}$$

with full-CI representations (S31)-(S33) of the electronic eigenfunctions Ψ_k, $k=0$, $1\pm$, $2\pm$. The coefficients $c_k = d_k e^{-i\delta_k}$ with amplitudes d_k and initial ($t=0$) phases δ_k depend on the $\pm\pm$ combinations of the circularly right ($+$) or left ($-$) polarized laser pulses. In any case, they are normalized,

$$\sum_k d_k{}^2 = 1. \tag{S55}$$

Since the wave function does not depend on an overall phase, we may set $\delta_0 = 0$ for the ground state $k=0$.

The corresponding many-electron probability densities are sums of time-independent (ti) and time-dependent (td) contributions which consist of three diagonal plus three off-diagonal terms,

$$\begin{aligned}\rho_{\pm\pm}^{(N)}(t) &= \Psi_{\pm\pm}^*(t)\Psi_{\pm\pm}(t) = \rho_{\pm\pm,\mathrm{ti}}^{(N)} + \rho_{\pm\pm,\mathrm{td}}^{(N)}(t) \\ &\equiv d_0^2\left|\Psi_0\right|^2 + d_{1\pm}^2\left|\Psi_{1\pm}\right|^2 + d_{2\pm}^2\left|\Psi_{2\pm}\right|^2 \\ &\quad + d_0 d_{1\pm}[\Psi_0^*\Psi_{1\pm}e^{-i(\delta_1+\omega_1 t)} + \Psi_0\Psi_{1\pm}^*e^{+i(\delta_1+\omega_1 t)}] \\ &\quad + d_0 d_{2\pm}[\Psi_0^*\Psi_{2\pm}e^{-i(\delta_2+\omega_2 t)} + \Psi_0\Psi_{2\pm}^*e^{+i(\delta_2+\omega_2 t)}] \\ &\quad + d_{1\pm}d_{2\pm}[\Psi_{1\pm}^*\Psi_{2\pm}e^{-i(\delta_2-\delta_1+\omega_2 t-\omega_1 t)} + \Psi_{1\pm}\Psi_{2\pm}^*e^{+i(\delta_2-\delta_1+\omega_2 t-\omega_1 t)}] \\ &\equiv d_0^2\rho_0^{(N)} + d_{1\pm}^2\rho_{1\pm}^{(N)} + d_{2\pm}^2\rho_{2\pm}^{(N)} \\ &\quad + d_0 d_{1\pm}\rho_{0,1\pm}^{(N)}(t) + d_0 d_{2\pm}\rho_{0,2\pm}^{(N)}(t) + d_{1\pm}d_{2\pm}\rho_{1\pm,2\pm}^{(N)}(t)\ .\end{aligned} \tag{S56}$$

The many-electron probability densities are normalized,

$$\int\!\ldots\!\int d\mathbf{q}_1\ldots d\mathbf{q}_N\,\rho_{\pm\pm}^{(N)}(t) = d_0^2 *1 + d_{1\pm}^2 *1 + d_{2\pm}^2 *1 = 1\ . \tag{S57}$$

This is a consequence of the normalization of the coefficients (S55) and the orthonormality of the eigenfunctions, eqn. (S36).

By analogy with eqn. (S40), the electronic densities in full-CI are obtained as the corresponding mean values of the density operator in eqn. (S39). Accordingly, they consist of a sum of a time-independent and a time-dependent contributions with corresponding three diagonal plus three off-diagonal terms

$$\begin{aligned}\rho_{\pm\pm}(\mathbf{r},t) &= \rho_{\pm\pm,\mathrm{ti}}(\mathbf{r}) + \rho_{\pm\pm,\mathrm{td}}(\mathbf{r},t) \equiv d_0^2\rho_0(\mathbf{r}) + d_{1\pm}^2\rho_{1\pm}(\mathbf{r}) + d_{2\pm}^2\rho_{2\pm}(\mathbf{r}) \\ &\quad + d_0 d_{1\pm}\rho_{0,1\pm}(\mathbf{r},t) + d_0 d_{2\pm}\rho_{0,2\pm}(\mathbf{r},t) + d_{1\pm}d_{2\pm}\rho_{1\pm,2\pm}(\mathbf{r},t)\ .\end{aligned} \tag{S58}$$

For the first three diagonal time-independent terms of eqn. (S58), we can adapt the results (S50) for the electronic densities of the eigenstates $1\ {}^1\Sigma^+$, $1\ {}^1\Pi_{\pm1}$ and $2\ {}^1\Pi_{\pm1}$. The time dependent off-

diagonal terms are evaluated by the same approach. For example, integration of the many-electron term $\Psi_0^*\Psi_{1\pm}$ in eqn. (S56) yields the contribution $d_0 d_{1\pm}\delta\rho_{0,1\pm}(r,t)e^{-i(\delta_1+\omega_1 t)}$ of $\rho_{0,1\pm}(r,t)$,

$$\begin{aligned}\delta\rho_{0,1\pm}(\mathbf{r},t) &= \sum_{j=1}^{N}\sum_{l_1<\ldots<l_N}\sum_{l_1^{'}<\ldots<l_N^{'}} C^{0}_{l_1,\ldots,l_N} C^{1\pm}_{l_1^{'},\ldots,l_N^{'}} \\ &\quad \int\ldots\int d\mathbf{q}_1\ldots d\mathbf{q}_N \left|\varphi_{l_1},\varphi_{l_2},\ldots,\varphi_{l_N}\right|^{*}\delta(\mathbf{r}_j-\mathbf{r})\left|\varphi_{l_1^{'}},\varphi_{l_2^{'}},\ldots,\varphi_{l_N^{'}}\right| \\ &= \sum_{j=1}^{N}\sum_{l_1<\ldots<l_N}\sum_{l_1^{'}<\ldots<l_N^{'}} C^{0}_{l_1,\ldots,l_N} C^{1\pm}_{l_1^{'},\ldots,l_N^{'}} \\ &\quad \int\ldots\int d\mathbf{q}_1\ldots d\mathbf{q}_N \varphi^{*}_{l_1}(\mathbf{q}_1)\ldots\varphi^{*}_{l_N}(\mathbf{q}_N)\delta(\mathbf{r}_j-\mathbf{r})\varphi_{l_1^{'}}(\mathbf{q}_1)\ldots\varphi_{l_N^{'}}(\mathbf{q}_N)\ .\end{aligned} \tag{S59}$$

Similar to eqn. (S49), the integration in eqn. (S59) can be simplified,

$$\int\ldots\int d\mathbf{q}_1\ldots d\mathbf{q}_N \left|\varphi_{l_1},\varphi_{l_2},\ldots,\varphi_{l_N}\right|^{*}\delta(\mathbf{r}_j-\mathbf{r})\left|\varphi_{l_1^{'}},\varphi_{l_2^{'}},\ldots,\varphi_{l_N^{'}}\right| = \psi^{*}_{m_j n_j}(r,z,\phi)\psi_{m_j^{'} n_j^{'}}(r,z,\phi)\,. \tag{S60}$$

Different from the previous case (S46), however, the angular momentum quantum numbers of the two sets of spin-orbitals for the 1 $^1\Sigma^+$ and the 1 $^1\Pi_{\pm 1}$ states must sum up to different values, namely

$$\sum_j m_j = 0\,,\ \sum_j m_j^{'} = \pm 1\ . \tag{S61}$$

This implies that the angular momentum quantum numbers of the spin-orbitals $\varphi_{l_j}(\mathbf{q}_j)$ and $\varphi^{'}_{l_j}(\mathbf{q}_j)$ must be different, namely

$$m_j^{'} = m_j \pm 1\ .$$

Inserting the explicit forms of the spin-orbitals (S12) then yields

$$\begin{aligned}\delta\rho_{0,1\pm}(\mathbf{r},t)e^{-i(\delta_1+\omega_1 t)} &= \frac{1}{2\pi}\sum_{j=1}^{N}\sum_{l_1<\ldots<l_N}\sum_{l_1^{'}<\ldots<l_N^{'}} C^{0}_{l_1,\ldots,l_N} C^{1\pm}_{l_1^{'},\ldots,l_N^{'}}\delta_{l_1,l_1^{'}}\ldots\delta_{l_{j-1},l_{j-1}^{'}}\delta_{l_{j+1},l_{j+1}^{'}}\ldots\delta_{l_N,l_N^{'}} \\ &\quad \chi_{m_j,n_j}(r_j,z_j)e^{-im_j\phi}\chi_{m_j\pm 1,n_j^{'}}(r_j,z_j)e^{i(m_j\pm 1)\phi}e^{-i(\delta_1+\omega_1 t)} \\ &= \frac{1}{2\pi}\sum_{j=1}^{N}\sum_{l_1<\ldots<l_N}\sum_{l_1^{'}<\ldots<l_N^{'}} C^{0}_{l_1,\ldots,l_N} C^{1\pm}_{l_1^{'},\ldots,l_N^{'}}\delta_{l_1,l_1^{'}}\ldots\delta_{l_{j-1},l_{j-1}^{'}}\delta_{l_{j+1},l_{j+1}^{'}}\ldots\delta_{l_N,l_N^{'}} \\ &\quad \chi_{m_j,n_j}(r,z)\chi_{m_j\pm 1,n_j^{'}}(r,z)e^{i(\pm\phi-\delta_1-\omega_1 t)}\ .\end{aligned} \tag{S62}$$

The time-dependent phase $-\delta_1-\omega_1 t$ in the expression (S56) for the many-electron probability density is thus supplemented by the angular phase $\pm\phi$ in the corresponding term for the electronic

density, yielding the total phase $\pm\phi-\delta_1-\omega_1 t$; this is an important intermediate result which has enormous consequences for the final result, cf. eqn. (S66) below.

Likewise, integration of the N-electron term $\Psi_0\Psi_{1\pm}^*$ in eqn. (S56) yields the complementary complex conjugate contribution $d_0 d_1 \delta\rho_{0,\pm1}(\mathbf{r},t)^* e^{i(\delta_1+\omega_1 t)}$ of $\rho_{0,\pm1}(\mathbf{r},t)$. Adding the two contributions yields the first time-dependent off-diagonal term

$$\begin{aligned}\rho_{0,1\pm}(\mathbf{r},t) &= \frac{1}{2\pi}\sum_{j=1}^{N}\sum_{l_1<\ldots<l_N}\sum_{l_1'<\ldots<l_N'} C^{0}_{l_1,\ldots,l_N} C^{1\pm}_{l_1',\ldots,l_N'} \delta_{l_1,l_1'}\ldots\delta_{l_{j-1},l_{j-1}'}\delta_{l_{j+1},l_{j+1}'}\ldots\delta_{l_N,l_N'} \\ &\quad \chi_{m_j,n_j}(r,z)\chi_{m_j\pm1,n_j'}(r,z) * 2\cos(\pm\phi-\delta_1-\omega_1 t) \\ &\equiv 2\rho_{0,1\pm}(r,z)\cos(\pm\phi-\delta_1-\omega_1 t)\ .\end{aligned} \tag{S63}$$

By analogy with the derivation for the first time dependent contribution (S63) to the electronic densities of the $1\ {}^1\Sigma^+ + 1\ {}^1\Pi_{\pm1} + 2\ {}^1\Pi_{\pm1}$ superposition states, we obtain for the second and third contributions

$$\begin{aligned}\rho_{0,2\pm}(\mathbf{r},t) &= 2\rho_{0,2\pm}(r,z)\cos(\pm\phi-\delta_2-\omega_2 t)\ , \\ \rho_{1\pm,2\pm}(\mathbf{r},t) &= 2\rho_{1\pm,2\pm}(r,z)\cos\left[\pm\phi-\delta_1-\omega_1 t-(\pm\phi-\delta_2-\omega_2 t)\right]\end{aligned} \tag{S64}$$

where

$$\begin{aligned}\rho_{0,2\pm}(r,z) &= \frac{1}{2\pi}\sum_{j=1}^{N}\sum_{l_1<\ldots<l_N}\sum_{l_1'<\ldots<l_N'} C^{0}_{l_1,\ldots,l_N} C^{2\pm}_{l_1',\ldots,l_N'} \delta_{l_1,l_1'}\ldots\delta_{l_{j-1},l_{j-1}'}\delta_{l_{j+1},l_{j+1}'}\ldots\delta_{l_N,l_N'} \\ &\quad \chi_{m_j,n_j}(r,z)\chi_{m_j\pm1,n_j'}(r,z)\ , \\ \rho_{1\pm,2\pm}(r,z) &= \frac{1}{2\pi}\sum_{j=1}^{N}\sum_{l_1<\ldots<l_N}\sum_{l_1'<\ldots<l_N'} C^{1\pm}_{l_1,\ldots,l_N} C^{2\pm}_{l_1',\ldots,l_N'} \delta_{l_1,l_1'}\ldots\delta_{l_{j-1},l_{j-1}'}\delta_{l_{j+1},l_{j+1}'}\ldots\delta_{l_N,l_N'} \\ &\quad \chi_{m_j\pm1,n_j}(r,z)\chi_{m_j\pm1,n_j'}(r,z)\ .\end{aligned} \tag{S65}$$

The time-dependent electronic density of the $1\ {}^1\Sigma^+ + 1\ {}^1\Pi_{\pm1} + 2\ {}^1\Pi_{\pm1}$ superposition states can be obtained by inserting eqns. (S63) - (S65) into (S58). It is a sum of time-independent and time-dependent contributions which consist of three diagonal plus three off-diagonal terms,

$$\begin{aligned}\rho_{\pm\pm}(r,z,\phi,t) &= \rho_{\pm\pm,\mathrm{ti}}(r,z)+\rho_{\pm\pm,\mathrm{td}}(r,z,\phi,t) \\ &\equiv d_0^2\rho_0(r,z)+d_{1\pm}^2\rho_{1\pm}(r,z)+d_{2\pm}^2\rho_{2\pm}(r,z) \\ &\quad +2d_0 d_{1\pm}\rho_{0,1\pm}(r,z)\cos(\pm\phi-\delta_1-\omega_1 t)+2d_0 d_{2\pm}\rho_{0,2\pm}(r,z)\cos(\pm\phi-\delta_2-\omega_2 t) \\ &\quad +2d_{1\pm}d_{2\pm}\rho_{1\pm,2\pm}(r,z)\cos\left[\pm\phi-\delta_1-\omega_1 t-(\pm\phi-\delta_2-\omega_2 t)\right]\ .\end{aligned} \tag{S66}$$

Integration of eqn. (S66) yields the total number of electrons,

$$N = \int_0^\infty rdr \int_{-\infty}^\infty dz \int_0^{2\pi} d\phi \rho_{\pm\pm}(r,z,\phi,t) = \int_0^\infty rdr \int_{-\infty}^\infty dz \int_0^{2\pi} d\phi \rho_{\pm\pm,\mathrm{ti}}(r,z) \ . \tag{S67}$$

The last eqn. (S67) is obtained using eqns. (S53), (S55). An important consequence of the equality (S67) is that the positive and negative contributions to the time-dependent part of the electronic density $\rho_{\pm\pm,\mathrm{td}}(r,z,\phi,t)$ compensate each other,

$$\int_0^\infty rdr \int_{-\infty}^\infty dz \int_0^{2\pi} d\phi \rho_{\pm\pm,\mathrm{td}}(r,z) = 0 \tag{S68}$$

This final result of this Section, eqn. (S66), is adapted as eqn. (2) in the main text. It sheds important light on the assignment of chirality or non-chirality of the electronic density. All time-independent contributions and also the time-dependent contribution for the special case of two laser pulses with the same $++$ or $--$ circular polarizations depend on r and z, but not on ϕ: they have an infinite number of vertical symmetry planes and many other symmetry elements, i. e. they have $C_{\infty\mathrm{v}}$ symmetry, i. e. they are achiral. In contrast, the first two terms of the time dependent contributions depend on r, z and ϕ. The ϕ-dependence is in the arguments of the two cosine-functions, $\cos(\pm\phi - \delta_1 - \omega_1 t)$ and $\cos(\pm\phi - \delta_2 - \omega_2 t)$, respectively. This breaks all symmetry elements, that means it causes C_1 symmetry and makes the electronic density chiral, except for the rare events at $t = T_n$ when the two arguments of the cosine functions are co-incidentally equal to zero or to integer ($= n$) multiples of π, for the same value of ϕ called ϕ_n: This leaves the electronic density with a mirror plane at the angle ϕ_n and makes it achiral at the exclusive instants T_n. Gratifyingly, if the third term of the time-dependent contribution depends on ϕ, then the argument of its cosine function is automatically also equal to zero or to $n\pi$, at the same time T_n and for the same angle ϕ_n. As a resume, the first two terms of the time dependent contributions to the electronic density are decisive: they make the electronic density chiral for all times, except at the rare events $t = T_n$.

Supplementary Note 3: Quantum dynamics simulations of the excitation of the oriented heteronuclear diatomic molecule from the ground state $1\ {}^1\Sigma^+$ to the $1\ {}^1\Sigma^+ + 1\ {}^1\Pi_{\pm 1} + 2\ {}^1\Pi_{\pm 1}$ superposition states by means of two circularly polarized laser pulses

The time-dependent electronic wavefunction $\Psi(t')$ of the laser driven oriented heteronuclear diatomic molecule AB, e. g. AB = NaK, is evaluated as solution of the electronic time-dependent Schrödinger equation (TDSE), using the semiclassical dipole approximation

$$i\hbar \frac{\partial}{\partial t'} \Psi_{\pm\pm}(t') = [H_{\mathrm{el}} - \boldsymbol{\varepsilon}_{\pm\pm}(t') \cdot \mathbf{d}] \Psi_{\pm\pm}(t') \ . \qquad \text{(S69)}$$

Here, H_{el} is the electronic Hamilton operator for fixed internuclear distance $R = R_{\mathrm{e}}$, $\mathbf{d}$ is the electronic dipole operator, and $\boldsymbol{\varepsilon}_{\pm\pm}(t')$ is the electric field of the laser pulses. The subscript "$\pm\pm$" refers to four combinations of the two circularly polarized laser pulses labeled "1" and "2"; each of them may have circular right (+) or left (−) polarizations. The electronic wavefunction $\Psi_{\pm\pm}(t')$ depends on the $\pm\pm$ combinations of the laser pulses. The two laser pulses propagate synchronously along the laboratory *z*-axis i. e. parallel to the molecular orientation. Specifically, $\boldsymbol{\varepsilon}_{\pm\pm}(t')$ consists of two components,

$$\boldsymbol{\varepsilon}_{\pm\pm}(t') = \varepsilon_x(t')\hat{\mathbf{e}}_x + \varepsilon_{y\pm\pm}(t')\hat{\mathbf{e}}_y$$

with amplitudes

$$\varepsilon_x(t') = \varepsilon_1 s_1(t') \cos(\omega_1 t' + \eta_1) + \varepsilon_2 s_2(t') \cos(\omega_2 t' + \eta_2) \ ,$$

$$\varepsilon_{y\pm\pm}(t') = \pm\varepsilon_1 s_1(t') \sin(\omega_1 t' + \eta_1) \pm \varepsilon_2 s_2(t') \sin(\omega_2 t' + \eta_2) \quad \text{(S70)}$$

We employ laser pulses with the same Gaussian shapes

$$s_1(t') = s_2(t') \equiv s(t') = e^{-t'^2/\tau^2} \qquad \text{(S71)}$$

and with the same duration τ, but with different field strengths $\varepsilon_1 < \varepsilon_2$ and with different resonant frequencies $\omega_1 = E_1/\hbar < \omega_2 = E_2/\hbar$, where E_1 and E_2 are the electronic energies of the degenerate excited states labeled $k = 1\pm$ and $k = 2\pm$, respectively. The carrier envelope phases are set to zero, $\eta_1 = \eta_2 = 0$. The laser pulses achieve maximum intensities

$$I_{\max,j} = \varepsilon_0 c \varepsilon_j^{\,2}, \ j = 1, 2 \qquad \text{(S72)}$$

at time $t' = 0$ this property is actually used to define the time-zero of t'.

To solve the TDSE (S69), we set

$$\Psi_{\pm\pm}(t') = \sum_k c_{k\pm\pm}(t')\Psi_k \tag{S73}$$

$$\Psi_{\pm\pm}(t' = t_i) = \Psi_0 \tag{S74}$$

where the sum $\sum_k$ is over all states $k = 0,\ 1+,\ 1-,\ 2+,\ 2-$. The initial value is $\Psi_{\pm\pm}(t') = \Psi_0$ at the beginning $(t' = t_i)$ of the laser pulses. This converts the TDSE (S69) into the set of time-dependent differential equations for the coefficients,

$$\begin{aligned} i\hbar \frac{dc_{j\pm\pm}(t')}{dt'} &= E_j c_{j\pm\pm}(t') - \boldsymbol{\varepsilon}_{\pm\pm}(t')\sum_k \mathbf{d}_{jk} c_{k\pm\pm}(t') \\ &= E_j c_{j\pm\pm}(t') - \varepsilon_x(t')\sum_k d_{x,jk} c_{k\pm\pm}(t') - \varepsilon_{y\pm\pm}(t')\sum_k d_{y,jk} c_{k\pm\pm}(t') \end{aligned} \tag{S75}$$

with transition dipole matrix elements

$$d_{x,jk} = \left\langle \Psi_j \left| d_x \right| \Psi_k \right\rangle,\ d_{y,jk} = \left\langle \Psi_j \left| d_y \right| \Psi_k \right\rangle\ . \tag{S76}$$

In practice, the matrix elements (S76) for j or $k = 1\pm$ or $2\pm$ are evaluated by decomposing the eigenfunctions into *x*- and *y*-components

$$\Psi_{1\pm} = \left(1/\sqrt{2}\right)\left(\Psi_{1x} \pm i\Psi_{1y}\right),\ \Psi_{2\pm} = \left(1/\sqrt{2}\right)\left(\Psi_{2x} \pm i\Psi_{2y}\right)\ , \tag{S77}$$

All the electronic properties are calculated by MOLPRO[2] using the same basis functions reported in (Ref. 3) with state-averaged CASSCF[4]. The results are

$$\begin{aligned} \left\langle \Psi_0 \left| d_x \right| \Psi_{1x} \right\rangle &= \left\langle \Psi_0 \left| d_y \right| \Psi_{1y} \right\rangle = 3.084\ ea_0\ , \\ \left\langle \Psi_0 \left| d_x \right| \Psi_{2x} \right\rangle &= \left\langle \Psi_0 \left| d_y \right| \Psi_{2y} \right\rangle = 1.198\ ea_0\ , \\ \left\langle \Psi_{1x} \left| d_x \right| \Psi_{2x} \right\rangle &= \left\langle \Psi_{1y} \left| d_y \right| \Psi_{2y} \right\rangle = 2.306\ ea_0\ , \end{aligned} \tag{S78}$$

in very good agreement with the results of (Ref. 5). All other permanent or transition dipole elements vanish, for symmetry reasons. The resulting coefficients $c_{k\pm\pm}(t')$ yield the populations

$$P_{k\pm\pm}(t') = \left|c_{k\pm\pm}(t')\right|^2\ . \tag{S79}$$

We employ the following laser parameters:

$$\begin{aligned} &\tau = 8\ \text{fs}\ , \\ &\varepsilon_1 = 1.26\times 10^8\ \text{V/m},\ \varepsilon_2 = 3.24\times 10^8\ \text{V/m} \\ &\omega_1 = 3.300\ \text{fs}^{-1},\ \omega_2 = 3.959\ \text{fs}^{-1}\ . \end{aligned} \tag{S80}$$

The values of the corresponding maximum intensities and the photon energies are

$$I_{\max,++} = I_{\max,--} = 5.26\times10^{10}\ \text{W/cm}^2,$$

$$I_{\max,+-} = I_{\max,-+} = 5.26\times10^{10}\ \text{W/cm}^2\ ,$$

$$\hbar\omega_1 = E_1 = 2.172\ \text{eV},\ \hbar\omega_2 = E_2 = 2.606\ \text{eV}\,. \tag{S81}$$

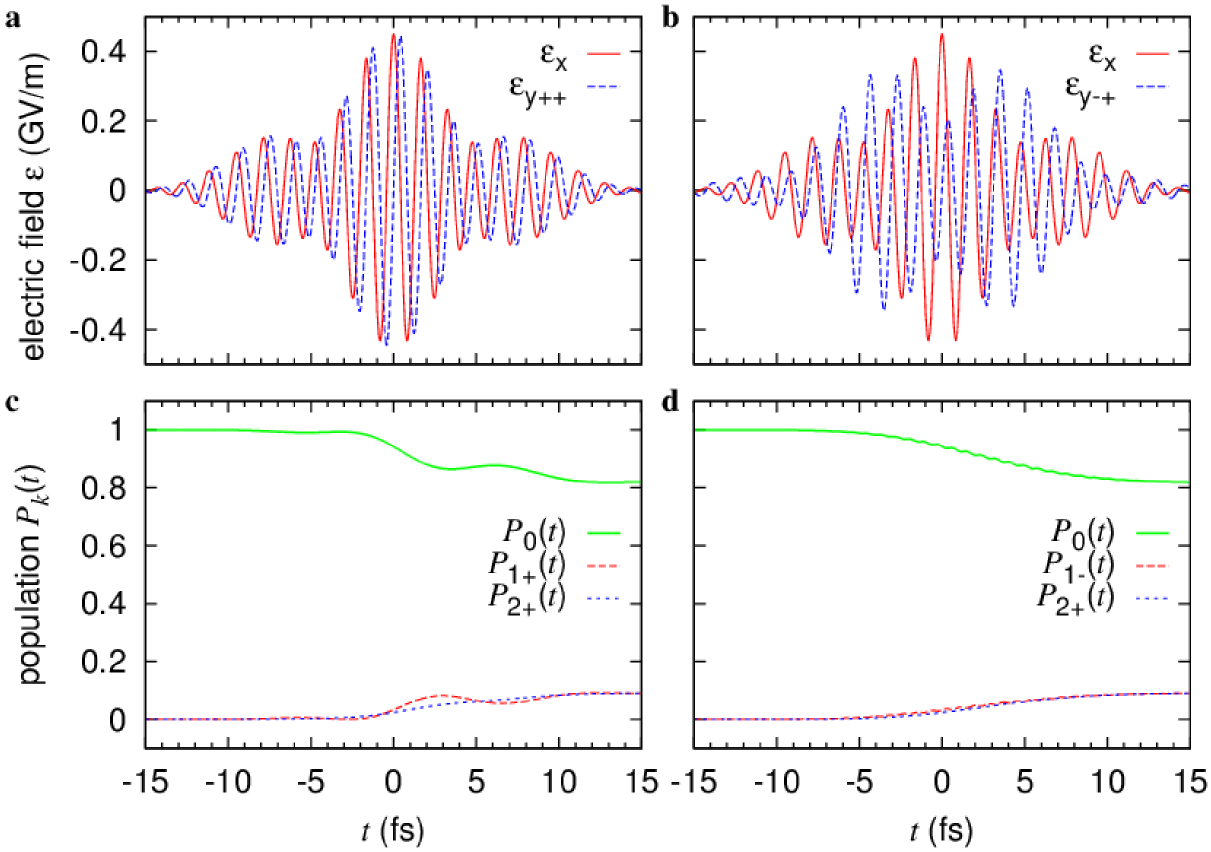

Supplementary Figure 4: Electric fields of two circularly polarized laser pulses for excitation of the oriented NaK molecule from the electronic ground state $1\ ^1\Sigma^+$ to the $1\ ^1\Sigma^+ + 1\ ^1\Pi_{+1} + 2\ ^1\Pi_{+1}$ (a) or $1\ ^1\Sigma^+ + 1\ ^1\Pi_{-1} + 2\ ^1\Pi_{+1}$ (b) superposition states, and the resulting population dynamics for states $1\ ^1\Sigma^+$ $(k=0)$, $1\ ^1\Pi_{+1}$ $(k=1+)$ or $1\ ^1\Pi_{-1}$ $(k=1-)$ and $2\ ^1\Pi_{+1}$ $(k=2+)$ (c), (d). Panels (a), (c) and (b), (d) are for two cases with the same $(++)$ or with opposite $(-+)$ polarizations. In panels (a) and (b), the *x*- and *y*-components of the electric fields are shown by red and blue lines. The laser parameters are the same for both cases, as specified in eqns. (S80), (S81).

As example, the corresponding electric fields of the circularly polarized laser pulses for two cases "$++$" and "$-+$" with the same (right, $+$ and again right, $+$) and with opposite (left, $-$ and

right, $+$) polarizations are shown in panels (a) and (b) of Supplementary Figure 4. The resulting population dynamics $P_k(t')$ are in panels (c) and (d), respectively. Equivalent results are obtained for all $\pm\pm$ combinations of the polarizations of the laser pulses. Apparently, the $P_k(t')$ approach asymptotic values for times $t' >\approx 12$ fs. For times $t' >\approx 12$ fs, the effect of the laser pulses is negligible. Hence it is reasonable to define the end of the pulses at time $t_e >\approx 12$ fs. For convenience, we set $t_e = 2\tau = 16$ fs; any other choice ≈ 12 fs $< t_e < 16$ fs would also do, without affecting any of the subsequent results. The time $t' = t_e$ is considered as the beginning of the (quasi-)field-free time evolution of the system, $t = 0$.

The parameters of the laser pulses (S80) are chosen to prepare the oriented NaK molecule in the $1\ {}^1\Sigma^+ + 1\ {}^1\Pi_{\pm1} + 2\ {}^1\Pi_{\pm1}$ superposition states with (quasi-)asymptotic target populations $P_0 = 0.82$, $P_{1\pm} = P_{2\pm} = 0.09$. For comparison, the results for the populations $P_k(t' = t_e) = |c_k(t' = t_e)|^2$, $k = 0$, $1\pm$, $2\pm$, obtained by the quantum dynamics simulations are listed in Supplementary Table 1. Supplementary Table 1 also has the amplitudes and the phases of the corresponding coefficients $c_{k\pm}(t = 0) = d_{k\pm} e^{-i\delta_{k\pm}}$.

Supplementary Table 1 | Results of the quantum dynamics simulations of the excitation of the oriented NaK from the ground state $1\ {}^1\Sigma^+$ to the $1\ {}^1\Sigma^+ + 1\ {}^1\Pi_{\pm1} + 2\ {}^1\Pi_{\pm1}$ superposition states by means of two circularly polarized laser pulses with right ($+$) or left ($-$) polarizations [a]

Case	P_0	$P_{1\pm}$	$P_{2\pm}$	d_0	$d_{1\pm}$	$d_{2\pm}$	$\delta_{1\pm}$	$\delta_{2\pm}$
$++/--$	0.820	0.090	0.090	0.905	0.300	0.300	0.907	-1.079
$-+/+-$	0.820	0.091	0.090	0.905	0.301	0.301	0.939	-1.076

[a] P_k, d_k and δ_k denote the populations, amplitudes and phases of the coefficients for states $k = 0$, ±1, ±2 at the end ($t' = t_e$) of the laser pulses.

Accordingly, we calculated the time-dependent electronic density, eqn. (2), for the four cases of $\pm\pm$ circular polarizations. The electronic densities are calculated using the ORBKIT package[6,7]. Representative snapshots are shown in Fig. 2 of the main text, Supplementary Figures 1-3.

Supplementary Note 4: Estimation of the decoherence time for the $1\ {}^1\Sigma^+ + 1\ {}^1\Pi_{\pm1} + 2\ {}^1\Pi_{\pm1}$ **superposition states of oriented NaK**

The present quantum dynamics simulations of the chirality flips of the electronic density of the oriented NaK molecule in $1\ {}^1\Sigma^+ + 1\ {}^1\Pi_{\pm1} + 2\ {}^1\Pi_{\pm1}$ superposition states prepared by two circularly polarized laser pulses are carried out for fixed bond length, $R_e\left(1\ {}^1\Sigma^+\right) = 3.499\,\mathring{A}$ [8,9]. This approximation of frozen nuclei is motivated by the fact that the periods of the chirality flips, i. e. $\Delta T_{++} = \Delta T_{--} = 4.764$ fs and $\Delta T_{-+} = \Delta T_{+-} = 0.433$ fs for preparations by laser pulses with the same ($++$ or $--$) or with opposite ($-+$ or $+-$) polarizations, are much shorter than the vibrational periods in electronic states $1\ {}^1\Sigma^+$, $1\ {}^1\Pi_{\pm1}$ and $2\ {}^1\Pi_{\pm1}$, with harmonic reference values (i. e. with lower limits) of $T_{\mathrm{vib}} = 269.5$ fs, 484.5 fs and 402.1 fs, respectively[3]. It is thus reasonable to assume that the nuclei stand practically still during the first chirality flip(s). This approximation implies coherent electron dynamics. The goal of this Section of SI is to support this approximation by an estimate of the time t_{dec} of decoherence of the electron dynamics due to nuclear motions[10].

For this purpose, the ansatz (S73) for the time-dependent electronic wavefunction $\psi_{\pm\pm}(t')$ is replaced by the Born-Huang expansion[11]

$$\Psi_{\pm\pm}(\mathbf{q}, R, t') = \sum_k \Psi_k(\mathbf{q}; R)\chi_{k\pm\pm}(R; t') \qquad \text{(S82)}$$

in terms of the coupled time-independent electronic wavefunctions $\Psi_k(\mathbf{q}; R)$ times time-dependent nuclear wavefunctions $\chi_{k\pm\pm}(R; t')$ which depend on the $\pm\pm$ combinations of the polarizations of the laser pulses. As in eqn. (S74), the sum $\sum_k$ in eqn. (S82) is over the electronic states $k = 0$, ±1, ±2. As before, the electronic eigenfunctions for the states k are solutions of the time independent electronic Schrödinger equation but now they are calculated not only for the frozen internuclear distance R_e but for arbitrary values of R. The electronic energies are written as potential energy curves $V_k(R)$ for the nuclear wavefunctions, depending on R. Instead of

converting the TDSE (S69) into the set of time-dependent differential equations for the coefficients (S75), now it is converted into the corresponding set of coupled nuclear TDSEs for the nuclear wavefunctions

$$\begin{aligned} i\hbar\frac{\partial}{\partial t'}\chi_{j\pm\pm}(R,t') &= \left[T_R + V_j\left(R\right)\right]\chi_{j\pm\pm}(R,t') - \sum_k \boldsymbol{\varepsilon}_{\pm\pm}(t')\cdot\mathbf{d}_{jk}(R)\chi_{k\pm\pm}(R,t') \\ &= \left[T_R + V_j\left(R\right)\right]\chi_{j\pm\pm}(R,t') - \sum_k \varepsilon_x(t')\cdot d_{x,jk}(R)\chi_{k\pm\pm}(R,t') - \varepsilon_{y\pm\pm}(t')\sum_k d_{y,jk}(R)\chi_{k\pm\pm}(R,t') \end{aligned} \tag{S83}$$

with nuclear kinetic energy operator

$$T_R = -\frac{\hbar^2}{2M}\frac{\partial^2}{\partial R^2} \tag{S84}$$

where $M = m_{\mathrm{Na}}m_{\mathrm{K}}/(m_{\mathrm{Na}}+m_{\mathrm{K}}) = 14.46$ u is the reduced mass. Eqns. (S83) account for the laser-dipole couplings which induce transitions between states j and k mediated by *R*-dependent transition dipole matrix elements

$$d_{x,jk}\left(R\right) = \left\langle \Psi_j\left(R\right)\middle| d_x \middle| \Psi_k\left(R\right)\right\rangle,\ d_{y,jk}\left(R\right) = \left\langle \Psi_j\left(R\right)\middle| d_y \middle| \Psi_k\left(R\right)\right\rangle\ , \tag{S85}$$

whereas non-adiabatic kinetic couplings are neglected.

The coupled nuclear TDSEs (S83) are solved numerically by means of the Split operator method[12]. The propagation starts at the beginning of the laser pulses ($t' = t_i$) when the oriented NaK is in the electronic and nuclear ground state, with nuclear ground state wavefunction $\chi_{k=0,0}(R)$. The eigenfunction $\chi_{k=0,0}(R)$ is evaluated as solution of the nuclear TISE

$$\left[T_R + V_{k=0}(R)\right]\chi_{00}(R) = E_{00}\chi_{00}(R)\ . \tag{S86}$$

It is normalized,

$$\left\langle \chi_{00} \middle| \chi_{00}\right\rangle_R = \int dR\chi_{00}^*(R)\chi_{00}(R) = 1\ . \tag{S87}$$

The initial conditions are

$$\chi_{0\pm\pm}(R,t'=t_i) = \chi_{00}(R)$$

and

$$\chi_{k\pm\pm}(R,t'=t_i) = 0 \text{ for } k \neq 0\ . \tag{S88}$$

In the present application, we set $t_i = -2\tau$. The resulting nuclear wavefunctions yield the probabilities of occupying electronic state k ,

$$P_{k\pm\pm}(t') = \langle \chi_{k\pm\pm}(t') | \chi_{k\pm\pm}(t') \rangle_R = \int dR \chi^*_{k\pm\pm}(R,t') \chi_{k\pm\pm}(R,t') \ . \quad \text{(S89)}$$

As a consequence of the initial normalization (S87), the probabilities are normalized,

$$\sum_k P_{k\pm\pm}(t') = 1 \ . \quad \text{(S90)}$$

The state-selective nuclear wavefunctions are, therefore, not normalized, but they can be re-normalized according to

$$\tilde{\chi}_{k\pm\pm}(R,t') = \chi_{k\pm\pm}(R,t') / \sqrt{P_{k\pm\pm}(t')} \ . \quad \text{(S91)}$$

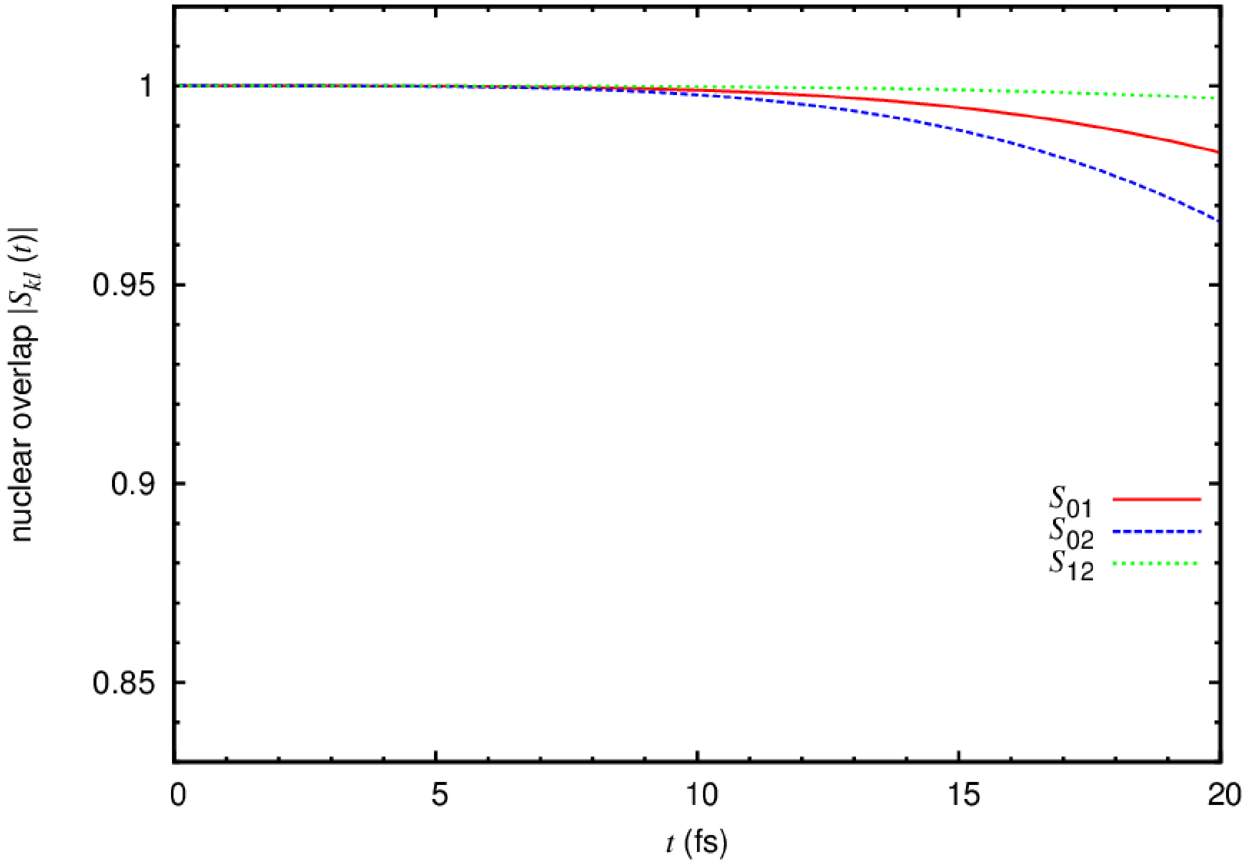

Supplementary Figure 5: Moduli of the overlaps $S_{kl}(t) = \langle \tilde{\chi}_k(t) | \tilde{\chi}_l(t) \rangle_{\mathrm{R}}$ of the re-normalized nuclear wavefunctions $\tilde{\chi}_k(t)$ and $\tilde{\chi}_l(t)$ propagated on potential energy surfaces $V_k(R)$ and $V_l(R)$ of NaK. The propagations start from the ground state wavefunction, $\tilde{\chi}_k(t=0) = \tilde{\chi}_l(t=0) = \chi_{00}$. The red, blue and green lines are for $kl = 01$, 02 and 12, respectively.

According to (Refs. 13-18), the decoherence time t_{dec} for field-free electron dynamics (i. e. independent of the $\pm\pm$ combinations of any laser pulses which launch the electron dynamics) is determined according to the decay time of the moduli $\left|\langle\tilde{\chi}_k(t')|\tilde{\chi}_l(t')\rangle_{\text{R}}\right|$ of the overlaps (or their squares) of the re-normalized nuclear wave functions

$$S_{k,l}(t') = \langle\tilde{\chi}_k(t')|\tilde{\chi}_l(t')\rangle_{\text{R}} \equiv \int dR\tilde{\chi}_k^*(R,t')\tilde{\chi}_l(R,t') \ . \tag{S92}$$

Typically, those propagations start from overlaps equal to 1 for the scenario of vertical excitations of the nuclear ground state wave to the excited states. In the present case, this corresponds to the overlaps $S_{k,l}(t) = \langle\tilde{\chi}_k(t)|\tilde{\chi}_l(t)\rangle_{\text{R}}$ of the nuclear wave functions $\tilde{\chi}_k(t)$ and $\tilde{\chi}_l(t)$ propagated on potential energies $V_k(R)$ and $V_l(R)$ for times t after the laser pulses ($t=0$ for $t'=t_e$), starting from

$$\chi_k(t=0) = \chi_l(t=0) = \chi_{00}(R) \ , \tag{S93}$$

irrespective of the $\pm\pm$ combinations of the laser pulses. The results are shown in Supplementary Figure 5. Apparently, the moduli of all the overlaps remain very close to 1, from $t=0$ to $t \approx 20$ fs and even beyond. That means that the electronic coherence lasts for more than 20 fs,

$$t_{\text{dec}} > 20 \text{ fs} \ . \tag{S94}$$

Our results for the chirality flips of the electronic densities in oriented NaK, yield periods $\Delta T_{++} = \Delta T_{--} = 4.764$ fs and $\Delta T_{-+} = \Delta T_{+-} = 0.433$ fs which are much shorter than t_{dec} . This confirms the validity of the underlying approximation of fixed nuclei.

Here we extend the previous estimates of the electronic decoherence times from field-free[14-18] to laser driven electron dynamics. Accordingly, the electronic decoherence depends on the decay time of the moduli $\left|\langle\tilde{\chi}_{k\pm\pm}(t')|\tilde{\chi}_{l\pm\pm}(t')\rangle_{\text{R}}\right|$ of the overlaps of the re-normalized nuclear wave functions driven by $\pm\pm$ combinations of two circularly polarized laser pulses with right $(+)$ and/or left $(-)$ polarizations,

$$S_{kl,\pm\pm}(t') = \langle\tilde{\chi}_{k\pm\pm}(t')|\tilde{\chi}_{l\pm\pm}(t')\rangle_{\text{R}} \equiv \int dR\tilde{\chi}_{k\pm\pm}^*(t')\tilde{\chi}_{l\pm\pm}(t') \ . \tag{S95}$$

The initial conditions (S92) imply the initial values

$$S_{kl,\pm\pm}(t'=t_i^+) = 1 \tag{S96}$$

immediately after the beginning of the laser pulses, denoted by " $t'=t_i^+$ ". As an example, Supplementary Figure 6 shows the time evolutions of the moduli of the overlaps $S_{kl,++}(t')$ for two circularly polarized laser pulses with the same (here: $++$) polarizations. Apparently, all values of $\left|S_{kl,++}(t'=t_e)\right|$ remain close to 1 at the end of the laser pulse. This confirms our model of quasi-coherent electron dynamics for fixed nuclei during the laser pulses which generate chirality with subsequent chirality flips of the electronic densities. Similar results are obtained for all $\pm\pm$ combinations of the laser pulses.

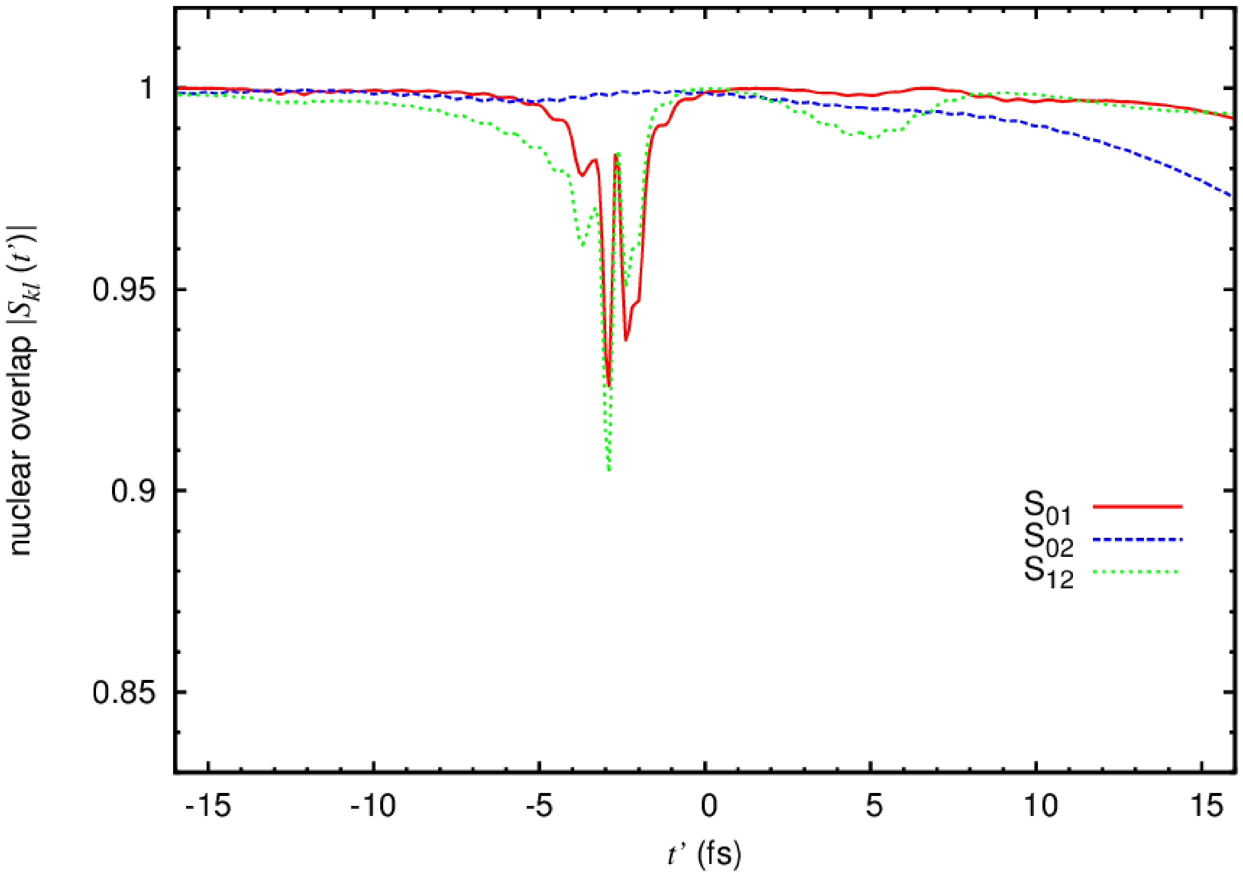

Supplementary Figure 6: Moduli of the overlaps $S_{kl,++}(t')=\left\langle\tilde{\chi}_{k++}(t')\middle|\tilde{\chi}_{l++}(t')\right\rangle_{\mathrm{R}}$ of the re-normalized nuclear wavefunctions $\tilde{\chi}_{k++}(t')$ and $\tilde{\chi}_{l++}(t')$ driven by two circularly polarized laser pulses with the same $(++)$ polarizations and propagated on potential energy surfaces $V_k(R)$ and $V_l(R)$ of NaK. The propagations start from the electronic and vibrational ground state specified in eqn. (S88). The red, blue and green lines are for $kl=01$, 02 and 12.

Supplementary References

1. Li, Y. & Li, C. Exact Analytical Form of Diatomic Molecular Orbitals. *ACS Omega.* **7**, 22594-22600 (2022).
2. Werner, H.-J., Knowles, P. J., Knizia, G., Manby, F. R. & Schütz, M. MOLPRO, version 2012.1, A package of ab initio programs. <heep://www.molpro.net>, 2012.
3. Bączek, K., Jasik, P., Kilich, T. & Sienkiewicz, J. E. Born-Oppenheimer potential energy curves of NaK from the optimized atomic basis sets. *Mol. Phys.* **120**, e2040628 (2022).
4. Knowles, P. J. & Werner, H.-J. An efficient second-order MC SCF method for long configuration expansions. *Chem. Phys. Lett.* **115**, 259-267(1985).
5. Aymar, A. & Dulieu, O. Calculations of transition and permanent dipole moments of heteronuclear alkali dimers NaK, NaRb and NaCs. *Mol. Phys.* **105**, 1733-1741 (2007).
6. Hermann, G., et al. Orbkit: A Modular python toolbox for cross-platform postprocessing of quantum chemical wavefunction data. *J. Comput. Chem.* **37**, 1511-1520 (2016).
7. Hermann, G., Pohl, V. & Tremblay, J. C. An open-source framework for analyzing n-electron dynamics. I. Multideterminantal wave functions. *J. Comput. Chem.* **38**, 1515-1527 (2017).
8. Gerdes, A., Hobein, M., Knöckel, H. & Tiemann, E. Ground state potentials of the NaK molecule. *Eur. Phys. J. D* **49**,67-73 (2008).
9. Russier-Antoine, I., Ross, A.-J., Aubert-Frecon, M., Martin, F. & Crozet, P. An improved potential energy curve for the ground state of NaK. *J. Phys. B: At. Mol. Opt. Phys.* **33**, 2753(2000).
10. Ma, H., Manz, J., Wang, H., Yan, Y. & Yang, Y. Ultrafast laser induced charge migration with de- and re-coherences in polyatomic molecules: A general method with application to pyrene. *J. Chem. Phys.* **158**, 124306 (2023).
11. Born, M. & Huang, K. "Appendix VIII" in Dynamical Theory of Crystal Lattices (Clarendon Press), pp. 406 (1954).
12. Leforestier, C., et al. A comparison of different propagation schemes for the time dependent Schrödinger equation. *J. Comput. Phys.* **94**, 59-80 (1991).

13. Scheidegger, A., Vaníček, J. & Golubev, N. Search for long-lasting electronic coherence using on-the-fly ab initio semiclassical dynamics. *J. Chem. Phys.* **156**, 034104 (2022).

14. Bandrauk, A. D., Chelkowski, S., Corkum, P. B., Manz, J. & Yudin, G. L. Attosecond photoionization of a coherent superposition of bound and dissociative molecular states: Effect of nuclear motion. *J. Phys. B: At. Mol. Opt. Phys.* **42**, 134001 (2009).

15. Vacher, M., Bearpark, M. J., Robb, M. A. & Malhado, J. P. Electron Dynamics upon Ionization of Polyatomic Molecules: Coupling to Quantum Nuclear Motion and Decoherence. *Phys. Rev. Lett.* **118**, 083001 (2017).

16. Arnold, C., Vendrell, O. & Santra, R. Electronic decoherence following photoionization: Full quantum-dynamical treatment of the influence of nuclear motion. *Phys. Rev. A* **95**, 033425 (2017).

17. Despré, V., Golubev, N. V. & Kuleff, A. I. Charge Migration in Propiolic Acid: A Full Quantum Dynamical Study. *Phys. Rev. Lett.* **121**, 203002 (2018).

18. Jia, D., Manz, J. & Yang, Y. De- and Recoherence of Charge Migration in Ionized Iodoacetylene. *J. Phys. Chem. Lett.* **10**, 4273-4277 (2019).
